# Inverse Association between Statin Use and Stomach Cancer Incidence in Individuals with Hypercholesterolemia, from the 2002–2015 NHIS-HEALS Data

**DOI:** 10.3390/ijerph17031054

**Published:** 2020-02-07

**Authors:** Hyo-Sun You, Nayoung You, Jae-Woo Lee, Hyoung-Ji Lim, Joungyoun Kim, Hee-Taik Kang

**Affiliations:** 1 Department of Family Medicine, Chungbuk National University Hospital, 776 1-Soonwhan-ro, Seowon-gu 28644, Cheongju, Korea; hyo920@gmail.com (H.-S.Y.); shrimp0611@gmail.com (J.-W.L.); hamultang@gmail.com (H.-J.L.); 2 National Cancer Center, 323 Ilsan-ro Ilsandong-gu Goyang-si, Gyoungki-do 28644, Korea; skdud5240@naver.com; 3 Department of Information & Statistics, Chungbuk National University, 1 Chungdae-ro, Seowon-gu, Cheongju, Chungbuk 28644, Korea; 4 Department of Family Medicine, Chungbuk National University College of Medicine, 1 Chungdae-ro, Seowon-gu, Cheongju, Chungbuk 28644, Korea

**Keywords:** HMG CoA reductase inhibitors, malignant neoplasms, stomach, hypercholesterolemia, incidence

## Abstract

*Purpose*: To investigate the association between statin use and stomach cancer incidence in individuals with hypercholesterolemia. *Materials and methods*: To examine the cumulative effect of statins, we defined a statin user as one who used statins during 2002–2003 at baseline. Statin users were further classified into high and low users according to the medication possession rate. Statin non-users consisted of participants who had never used statins during the entire period of 2002–2015, despite having hypercholesterolemia (total cholesterol level ≥250 mg/dL at baseline). Ultimately, 17,737 statin users and 13,412 statin non-users were used in the analysis. We performed survival analyses, considering the diagnosis of stomach cancer as an event of interest. *Results*: Median follow-up duration was 12.9 years. The cumulative incidence rates of stomach cancer were lowest in high users (1.90% in men and 0.98% in women). Compared to non-users, hazard ratios (95% confidential intervals) for stomach cancer of low users and high users were 0.953 (0.755–1.203) and 0.526 (0.399–0.693) in men and 0.629 (0.457–0.865) and 0.370 (0.256–0.535) in women, respectively, after adjusting for possible confounders. *Conclusions*: We observed an inverse association between statin use and stomach cancer incidence in participants with hypercholesterolemia.

## 1. Introduction

Malignant neoplasms are the number one cause of death in Korea. The incidence of cancer has steadily increased over time, although it has leveled off in recent years. Statistics Korea reports that an overwhelming number of Koreans die from malignant neoplasms, especially among 40-year-olds [1]. The age-standardized incidence rates of cancers in Korea were 301.2 in men and 266.1 in women per 100,000 persons in 2015 [1], which is similar to or higher than those of other developed countries (USA, 347.0 in men and 297.4 in women; UK, 284.0 in men and 267.3 in women; Japan, 260.5 in men and 185.7 in women) [2]. The best way to reduce cancer mortality is prevention and early detection. Health authorities in Korea provide a national cancer screening program for the five most common cancer types: stomach, liver, colorectum, breast, and uterine cervix [1]. Early diagnosis of stomach cancer may contribute to better survivor outcomes. If agents commonly prescribed for other indications can prevent cancer development, progression, and mortality, the burden of illness of malignant neoplasms would be significantly reduced.

Dyslipidemia results in atherosclerotic cardio-cerebrovascular diseases (CCVDs), which are the second and third highest causes of death in Korea [3]. Statins inhibit 3-hydroxy-3-methylglutaryl coenzyme A (HMG-CoA) reductase, which are commonly used for primary and secondary prevention of CCVDs in individuals with dyslipidemia. In addition to a lipid-lowering effect, statins have pleiotropic effects, including anti-inflammatory, immunomodulatory, and anti-oxidative properties. In case of cancer, some animal studies have shown conflicting results, finding carcinogenic effects or chemo-preventive properties [4,5,6]. These multipotent effects can act differently on various cancers such as breast cancer, colorectal, prostate, and lung cancer [7]. Additionally, these pleiotropic effects seem to be associated with the mimicking role of statin and glucose-6-phosphate dehydrogenase (G6PD) deficiency. G6PD deficiency is related to reduction of cardiovascular mortality but no evidence of lowering cancer risk [8,9]. Although there are some observational studies regarding the association between statin use and stomach cancer development [10,11,12], investigation in larger populations and for longer durations is needed.

The aim of this study was to investigate the association between statin use and incident malignant neoplasm of the stomach using the Korean National Health Insurance Service (NHIS)-National Health Screening Cohort (NHIS-HEALS) after adjusting for possible confounding factors. 

## 2. Methods

### 2.1. Study Participants

This study is based on the HEALS Database provided by the Korean NHIS [13]. NHIS-HEALS is a research database constructed in a cohort format to analyze the medical use and health outcome of screening tests. NHIS-HEALS consists of 514,795 people, randomly selected from those who received a health screening (*n* = 5,151,000) in 2002–2003, with an age between 40 and 79 years, and who also held health insurance as of the end of December 2002. The data have been anonymized. NHIS-HEALS provides insurance status, income information (socio-economic variables), information on hospital utilization, health checkups, and medical institution information during 2002–2013.

In this study, only individuals with hypercholesterolemia were included, based on the following criteria: (1) total cholesterol level ≥250 mg/dL at initial screening or (2) prescription of anti-dyslipidemia drugs including statins and fibric acid derivatives during 2002–2003. Among the 76,372 participants who met these criteria, we then excluded the following criteria. In case of diagnosed cancer or death in the first year of the follow up period (at 2004), we considered that participants already had the cause of death or cancer before the follow up started. So, we excluded subjects diagnosed with malignant neoplasm or who died in 2004.

(1) Participants diagnosed with malignant neoplasm with an International Classification of Diseases (ICD-10) code of C00–C97 or D00–D09 between January 2002 and December 2004;
(2) Participants who died from any cause between January 2002 and December 2004;
(3) Participants diagnosed with ischemic heart diseases (ICD-10 code I20–I25) or cerebrovascular diseases (ICD-10 code I60–I69) between January 2002 and December 2003;
(4) Participants aged 79 years or older at the time of the initial screening between January 2002 and December 2003.

**Figure 1:**
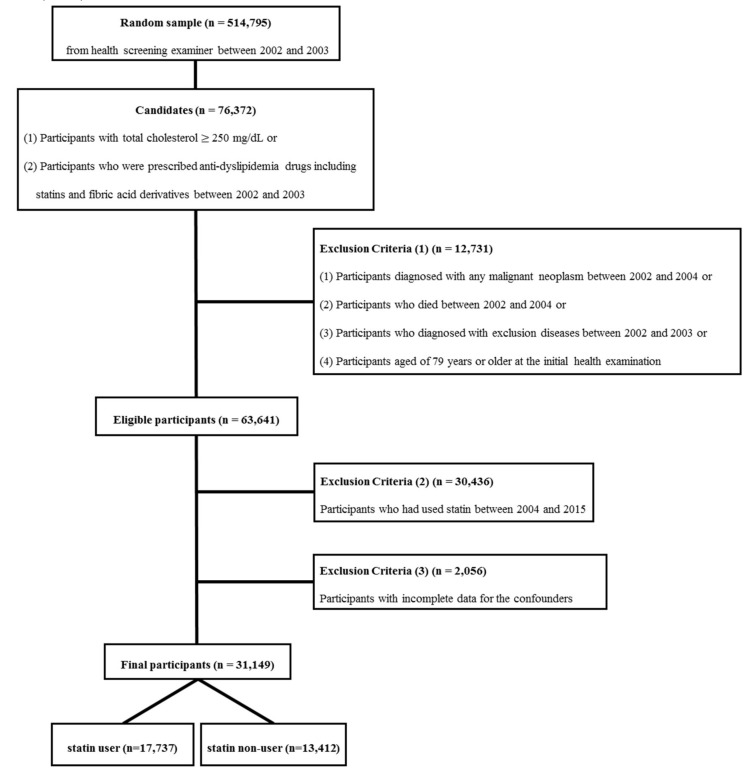
Flowchart of inclusion and exclusion criteria.

After applying the above exclusion criteria, 63,641 individuals were included. To investigate the effect of statins on the incidence of stomach cancer, we compared two groups: statin users and non-users. To examine the cumulative effect of statin usage, we defined statin users as those who used statins during 2002–2003, the early period of this study. In contrast, the statin non-user group consisted of participants who had never used statins during the entire period of 2002–2015, despite having hypercholesterolemia (total cholesterol level ≥250 mg/dL at initial screening). To meet these restrictions, we dropped participants (*n* = 30,436) who initiated statin use after January 1, 2004. In addition, as possible confounders, we considered age, body mass index (BMI), blood pressure, blood glucose levels, total cholesterol levels, smoking status, alcohol consumption, physical activity, and past medical history of hypertension and diabetes. Participants with incomplete data for these confounders (*n* = 2056) are dropped as well. Ultimately, 31,149 individuals (16,588 men and 14,561 women) were included in this analysis, with 17,737 statin users (8100 men and 9637 women) (Figure 1). The Institutional Review Board of Chungbuk National University approved the present study (CBNU-201711-BMETC-564-01), which was conducted according to the guidelines of the Declaration of Helsinki (1975).

### 2.2. Variables

We used values of confounding factors which were measured at the time of screening in 2002–2003. For participants screened in both 2002 and 2003, we used the older record from 2002. For continuous variables like age, blood glucose level, total cholesterol level, and blood pressure, we used the values as measured. Other variables were categorized. BMI (Body Mass Index) was originally continuous, but was then dichotomized into three groups (<23 kg/m^2^, 23–25 kg/m^2^, ≥25 kg/m^2^). For smoking status, participants were categorized into never smokers and ever smokers. Ever smokers included former and current smokers. Alcohol consumption was classified into three groups as follows: Rare, less than twice per month; Sometimes, twice per month to twice per week; Often, more than twice per week. Physical activity was divided into three groups: Rare, rarely incorporated exercise; Sometimes, between once and four times per week; Regular, more than four times per week. We considered history of hypertension and diabetes as binary variables with self-reported presence or absence according to a questionnaire. Information about income was expressed as an income percentile, and we categorized economic status according to individual income into three groups: Low, 0–30th percentile; Middle, 3st–70th percentile; High, 71st–100th percentile. 

### 2.3. Statin Use Assessment

Statin users were defined as participants who were prescribed statins during 2002–2003. The statins prescribed during the study period were pravastatin, simvastatin, atorvastatin, cerivastatin, lovastatin, and fluvastatin. The statin user group was subdivided into two groups according to the degree of statin usage, and we investigated the possible association with the incidence of stomach cancer. The degree of statin usage was evaluated based on the medication possession ratio (MPR), the ratio of the total prescription days of statins out of the total study period. Based on the sample median MPR, statin users were divided into low and high users. To compute the MPR, we needed to specify the research period, which varied by participant. For statin users, the start date was defined as the first prescription date for statin. For statin non-users, the start date was defined as the earlier date of screening with total cholesterol level above 250 mg/dL and the initial prescription date for other anti-dyslipidemic drugs, except statin. If the participants had stomach cancer during the follow-up, the date of the first diagnosis of stomach cancer was defined as the end date. For the participants who died without stomach cancer, the date of death was defined as the end date. If neither the stomach cancer nor the death occurred, the study end date is the later of the three days: 1) the last screening date, 2) the last clinic or hospital visit date, and 3) the last date of statin prescription. 

### 2.4. Diagnosis of Stomach Cancer

In this study, stomach cancer was defined as the main diagnosis with an ICD-10 code C16.0–C16.9 during 2005–2015 for malignant neoplasm of the stomach in order to exclude uncertain diagnoses. NHIS investigates and manages rare and incurable diseases such as malignant neoplasms to reduce the economic burden of patients with them. By restricting our analysis to a main diagnosis ICD-10 code of C16.0–C16.9, we reduced the possibility of a false positive for unconfirmed stomach cancer.

### 2.5. Statistical Analysis

Baseline characteristics of study participants are presented as the number (%) or mean ± standard error (SE) as appropriate for each variable. To evaluate heterogeneity by statin usage level, group comparisons were performed using the chi-squared test for categorical variables and ANOVA for continuous variables. To see the effects of statin usage on stomach cancer, we measured the time from screening to the initial diagnosis of stomach cancer and then treated it as survival data. Other than the diagnosis of stomach cancer, nothing could be an event but a censoring. To compare the stomach cancer rate over time depending on statin usage level, we performed the survival analysis. To adjust for the effects of possible confounders, Cox proportional hazard regression models were employed. P-values and 95% confidence intervals (CIs) are presented along with the estimates of hazard ratios (HRs). All P-values are two-sided, and P-values less than 0.05 were considered statistically significant. The statistical package SAS enterprise guide version 7.1 (SAS^®^ Institute Incorporation, Cary, NC, U.S.) and R^®^ studio version 3.3.3 (Fundation for Statistical Computing, Viena, Austria) were used to perform the analyses in this study.

## 3. Results

Median follow-up duration was 12.9 years. Table 1 summarizes all participant characteristics by sex. The mean age was 52.6 years in men and 58.7 years in women. Table 2 shows baseline characteristics according to statin use. Individuals on anti-dyslipidemic medication were older in both sexes. Statin users had higher BMI, systolic blood pressure, and glucose levels, but lower total cholesterol levels. Statin users tended to have more diabetes mellitus and hypertension. In general, statin users participated more in regular physical activity and belonged to a higher economic status.

Results from Cox proportional hazards regression models are provided in Table 3 for men and women. Compared to non-users, HRs (95% CIs) for low users were 0.922 (0.741–1.149) in men and 0.656 (0.486–0.885) in women after adjusting for age only (Model 1). The HRs (95% CIs) for high users were 0.521 (0.402–0.676) in men and 0.395 (0.280–0.559) in women. After fully adjusting for potential confounding factors, HRs (95% CIs) of low users and high users were 0.953 (0.755–1.203) and 0.526 (0.399–0.693) in men and 0.629 (0.457–0.865) and 0.370 (0.256–0.535) in women, respectively (Model 3), implying a lower risk for stomach cancer in statin users (especially high users) than in non-users.

Figure S1 shows the survival rates of stomach cancer according to statin usage based on the Cox proportional hazards regression model. For the plot, for each confounding factors except for the statin usage, each mean value was inserted in the Cox model. In both men and women, high statin users had the lowest incidence of stomach cancer. For women, in particular, the incidence rates of stomach cancer increased as statin use moved from high to low to none.

## 4. Discussion

The scientific debate on the association between statin use and the risk of malignant neoplasm has been inconclusive. In this study, we found that statin users with hypercholesterolemia were at lower risk for stomach cancer than non-users. 

Statins are the most prescribed drugs and have pluripotent beneficial effects including lipid-lowering action. Statins inhibit HMG-CoA reductase in the mevalonate pathway. In the lower mevalonate pathway, isoprenoid molecules are the building blocks of cholesterol, dolichol, and coenzyme Q10 [14]. Intermediate products of this pathway include farnesyl pyrophosphate and geranylgeranyl pyrophosphate, which play important roles in cell signaling and proliferation through other signaling proteins such as Ras and Rho [15]. In addition, the potential effect of statins against carcinogenesis has been considered as derived from modulation of the insulin-like growth factor pathway [10]. Although a few studies have reported that statins are carcinogenic in animal models [6], more evidence supports a chemo-preventive role for cancers [16,17]. These chemo-preventive effects of statins result from enhancing apoptosis, suppressing angiogenesis, inhibiting low-grade chronic inflammation, and changing tumor microenvironments, in addition to effects downstream of the mevalonate pathway [18,19,20,21].

Several studies have found that statins have protective effects against stomach cancer development [10,11,12], while others failed to find a significant association [22,23]. Chiu et al. suggested that statins might reduce the incidence of stomach cancer based on a population-based case-control study in Taiwan [12]. Lee et al. reported a strong inverse relationship between statin use and gastric adenocarcinoma in Korean diabetic patients after matching by age and sex [10]. A meta-analysis by Wu et al. indicated that statin use was significantly related to a 27% risk reduction of stomach cancer, although a neutral effect was shown in randomized controlled trials (RR [95% CIs] = 0.84 [0.61–1.14]) [11]. In contrast, Graaf et al. observed that statin therapy was not associated with stomach cancer risk but was related to an overall lower risk of cancer [22]. Kouppala et al. demonstrated that statins had no short-term anti-cancer effect in a meta-analysis [24], failing to prove an association between statin use and stomach cancer risk. They suggested a long-term study to validate this association. Statin exposure in rodents at levels close to human doses may result in cancer [6]. In this study, we found that statin usage in the real world was prospectively associated with a risk reduction of stomach cancer development over a relatively longer duration (median follow-up duration 12.9 years). In addition, there was a dose response correlation. 

Our study has advantages that distinguish it from other studies. We used data from a large population provided by the NHIS, based on real world measurements in the clinical setting. Thus, this present study represents the entire Korean population. Korean public authorities recommend obligatory medical insurance to cover the entire Korean population, including individuals of low socioeconomic status, and almost all enroll in national health insurance. The participation rate for regular employees in Korea was 97% in 2013. Thus, national health insurance claim data include diagnosis, blood tests, and prescriptions of most of the Korean population. In addition, the Korean Ministry of Health and Welfare provides all adults over the age of 40 free health checkup services, including health and lifestyle questionnaires, some blood tests such as lipid profiling, and screening for the five most common types of malignant neoplasms, including stomach cancer. In particular, the national cancer screening program recommends biennial gastric endoscopic examination or an upper gastro-intestinal series to detect stomach cancer early in all adults more than 40 years old. All data from the national health checkup service and private medical sectors are under the control of the Ministry of Health and Welfare in order to check and pay insurance claims from medical institutions. Because almost all data from questionnaires, blood samples, prescriptions, and diagnosis from NHIS-HEALS are real-world data, recall bias and misclassification are minimized. Furthermore, special diseases such as malignant neoplasms and rare diseases such as rheumatism are more strictly monitored by the Korean NHIS, because individuals with these diseases spend less out-of-pocket for medical expenses than with other common diseases due to insurance reimbursement. For these reasons, the possibility of a false positive diagnosis and misclassification of stomach cancer is extremely low. To control for health inequity based on socioeconomic status, we adjusted for individual income, because socioeconomic status is a major indicator of healthcare accessibility. We previously reported that cancer screening rates within the last two years were 56.6% in men and 66.0% in women in the Korean population [25]. More individuals with a high socioeconomic status participate in overall cancer screenings than those with low socioeconomic status. Participation in more cancer screenings increases the likelihood of incidental cancer diagnosis. However, the incidence of stomach cancer was low in this study even among statin users with a high socioeconomic status, suggesting that statin usage may reduce the incidence of stomach cancer.

There are several limitations which should be kept in mind. First, although we adjusted for several potential confounding factors, we were unable to completely control for others, such as lifestyle factors and underlying genetic or familial conditions. Despite exclusion of underlying CCVDs at baseline, statin users have more diabetes and hypertension and a better lifestyle in terms of cigarette smoking, alcohol consumption, and physical activity. A less healthy lifestyle can have a negative effect on the development of cancer despite statistical controls. However, more diabetic patients who use statins also have a higher risk for stomach cancer [10]. Second, we could not discriminate between the types of statin (lipophilic, hydrophilic, or mixed). Previous studies report that statin types can differently affect the development, progression, and mortality of malignant neoplasms [26,27,28]. Third, we could not verify that statin users took their medications as prescribed. In order to minimize misclassification, we used the MPR, which correlates well with patient adherence [29]. Fourth, there was limited available information about risk factors for stomach cancer, including helicobacter pyloric infection and peptic ulcer diseases, because the NHIS-HEALS database does not provide such information. Fifth, as a risk factor for stomach cancer, dietary habit also limited information. Previous studies have shown that fresh vegetables and fruits decreased risk while salty food, high-carbohydrate, and charcoal grilled meat increased risk of gastric cancer [30,31]. However, this information also was not contained in the database and could not be addressed.

## 5. Conclusions

In conclusion, there was an inverse association between statin use and stomach cancer incidence in individuals with hypercholesterolemia. This finding suggests that active statin therapy in individuals with hypercholesterolemia can reduce the development of stomach cancer.

## Figures and Tables

**Table 1:** Participant characteristics.

	Men	Women
Number	16,588	14,561
Age, years	52.6 ± 0.1	58.7 ± 0.1
Body Mass Index, kg/m^2^	24.9 ± 0.0	25.0 ± 0.0
Systolic Blood Pressure, mmHg	131.5 ± 0.1	131.0 ± 0.2
Glucose, mg/dL	108.1 ± 0.4	105.7 ± 0.4
Total cholesterol, mg/dL	247.1 ± 0.4	247.0 ± 0.4
Diabetes Mellitus, %	7.8 ± 0.2	9.6 ± 0.2
Hypertension, %	11.8 ± 0.3	20.3 ± 0.3
Ever smokers, %	58.6 ± 0.4	4.2 ± 0.2
Drinking status, %		
Rare	34.3 ± 0.4	85.8 ± 0.3
Sometimes	45.9 ± 0.4	12.6 ± 0.3
Often	19.8 ± 0.3	1.7 ± 0.1
Physical activity, %		
Rare	47.2 ± 0.4	65.1 ± 0.4
Sometimes	42.6 ± 0.4	23.6 ± 0.4
Regular	10.2 ± 0.2	11.3 ± 0.3
Economic status, %		
Low	18.6 ± 0.3	27.5 ± 0.4
Middle	31.4 ± 0.4	32.4 ± 0.4
High	50.1 ± 0.4	40.1 ± 0.4

Drinking status: Rare, less than twice per month; Sometimes, twice per month–twice per week; Often, more than twice per week. Physical activity: Rare, less than once per week; Sometimes, once–four days per week; Regular, more than four day per week. Economic status: Low, 0–30th percentile of income; Middle, 31st–70th percentile of income; High, 71st–100th percentile of income.

**Table 2:** Baseline characteristics according to statin usage.

**Male**	**Non-Users**	**Low Users**	**High Users**	**P-value**
Number	8488	4050	4050	NA
Age, years	51.2 ± 0.1	53.4 ± 0.1	54.5 ± 0.1	<0.001
Body Mass Index, kg/m^2^	24.5 ± 0.0	25.0 ± 0.0	25.5 ± 0.0	<0.001
Systolic Blood Pressure, mmHg	129.6 ± 0.2	132.0 ± 0.3	134.9 ± 0.3	<0.001
Glucose, mg/dL	104.4 ± 0.6	107.8 ± 0.7	116.1 ± 0.8	<0.001
Total cholesterol, mg/dL	263.2 ± 0.5	226.6 ± 0.7	234.0 ± 0.8	<0.001
Diabetes Mellitus, %	2.9 ± 0.2	9.3 ± 0.5	16.3 ± 0.6	<0.001
Hypertension, %	5.0 ± 0.2	14.5 ± 0.6	23.6 ± 0.7	<0.001
Ever smokers, %	61.8 ± 0.5	55.7 ± 0.8	54.8 ± 0.8	<0.001
Drinking status, %				<0.001
Rare	32.9 ± 0.5	34.9 ± 0.7	36.6 ± 0.8	
Sometimes	46.7 ± 0.5	44.7 ± 0.8	45.3 ± 0.8	
Often	20.4 ± 0.4	20.3 ± 0.6	18.1 ± 0.6	
Physical activity, %				<0.001
Rare	48.4 ± 0.5	48.2 ± 0.8	43.3 ± 0.8	
Sometimes	43.0 ± 0.5	41.2 ± 0.8	43.2 ± 0.8	
Regular	8.4 ± 0.3	10.6 ± 0.5	13.5 ± 0.5	
Economic status, %				<0.001
Low	20.6 ± 0.4	17.1 ± 0.6	15.9 ± 0.6	
Middle	32.2 ± 0.5	31.8 ± 0.7	28.8 ± 0.7	
High	47.2 ± 0.5	51.1 ± 0.8	55.3 ± 0.8	
**Female**	**Non-User**	**Low User**	**High User**	**P-value**
Number	4924	4818	4819	NA
Age, years	58.2 ± 0.1	58.7 ± 0.1	59.3 ± 0.1	<0.001
Body Mass Index, kg/m^2^	24.3 ± 0.0	25.2 ± 0.0	25.4 ± 0.0	<0.001
Systolic Blood Pressure, mmHg	128.6 ± 0.3	131.1 ± 0.3	133.3 ± 0.3	<0.001
Glucose, mg/dL	103.7 ± 0.9	104.6 ± 0.6	108.8 ± 0.7	<0.001
Total cholesterol, mg/dL	266.9 ± 0.6	234.4 ± 0.7	239.3 ± 0.7	<0.001
Diabetes Mellitus, %	3.2 ± 0.3	10.0 ± 0.4	15.7 ± 0.5	<0.001
Hypertension, %	9.9 ± 0.4	20.6 ± 0.6	30.5 ± 0.7	<0.001
Ever smokers, %	4.7 ± 0.3	4.0 ± 0.3	3.9 ± 0.3	0.092
Drinking status, %				<0.001
Rare	83.5 ± 0.5	85.5 ± 0.5	88.4 ± 0.5	
Sometimes	14.4 ± 0.5	12.8 ± 0.5	10.4 ± 0.4	
Often	2.0 ± 0.2	1.7 ± 0.2	1.2 ± 0.2	
Physical activity, %				<0.001
Rare	70.3 ± 0.7	65.7 ± 0.7	59.3 ± 0.7	
Sometimes	20.4 ± 0.6	23.0 ± 0.6	27.6 ± 0.6	
Regular	9.3 ± 0.4	11.4 ± 0.5	13.1 ± 0.5	
Economic status, %				<0.001
Low	31.6 ± 0.7	27.8 ± 0.6	23.1 ± 0.6	
Middle	31.8 ± 0.7	33.6 ± 0.7	31.9 ± 0.7	
High	36.6 ± 0.7	38.6 ± 0.7	44.9 ± 0.7	

Drinking status: Rare, less than twice per month; Sometimes, twice per month–twice per week; Often, more than twice per week. Physical activity: Rare, less than once per week; Sometimes, once–four days per week; Regular, more than four day per week. Economic status: Low, 0–30th percentile of income; Middle, 31st–70th percentile of income; High, 71st-100th percentile of income.

**Table 3:** Cox proportional hazard regression for stomach cancer incidence.

Hazard Ratios (95% Confidence Intervals)	Male	Female
Model 1		
Low users vs Non-users	0.922 (0.741–1.149)	0.656 (0.486–0.885)
High users vs Non-users	0.521 (0.402–0.676)	0.395 (0.280–0.559)
Model 2		
Low users vs Non-users	0.930 (0.746–1.158)	0.657 (0.486–0.887)
High users vs Non-users	0.532 (0.410–0.690)	0.397 (0.280–0.562)
Model 3		
Low users vs Non-users	0.953 (0.755–1.203)	0.629 (0.457–0.865)
High users vs Non-users	0.526 (0.399–0.693)	0.370 (0.256–0.535)

Model 1: adjusted for age. Model 2: adjusted for smoking status (ever and never smokers), drinking status (rare, sometimes, and often), and physical activity (rare, sometimes, and regular) in addition to Model 1. Model 3: adjusted for body mass index, systolic blood pressure, total cholesterol, economic status (low, middle, and high), diabetes (yes or no), and hypertension (yes or no) in addition to Model 2.

## Supplementary Materials

The following are available online at https://www.mdpi.com/1660-4601/17/3/1054/s1. Figure S1: Survival rates of stomach cancer according to statin usage.
