# Supplementary figures and images for: Inverse Association between Statin Use and Stomach Cancer Incidence in Individuals with Hypercholesterolemia, from the 2002–2015 NHIS-HEALS Data
Source: Int J Environ Res Public Health. 2020 Feb 7;17(3):1054. doi: 10.3390/ijerph17031054 (PMC7037780; doi:10.3390/ijerph17031054)

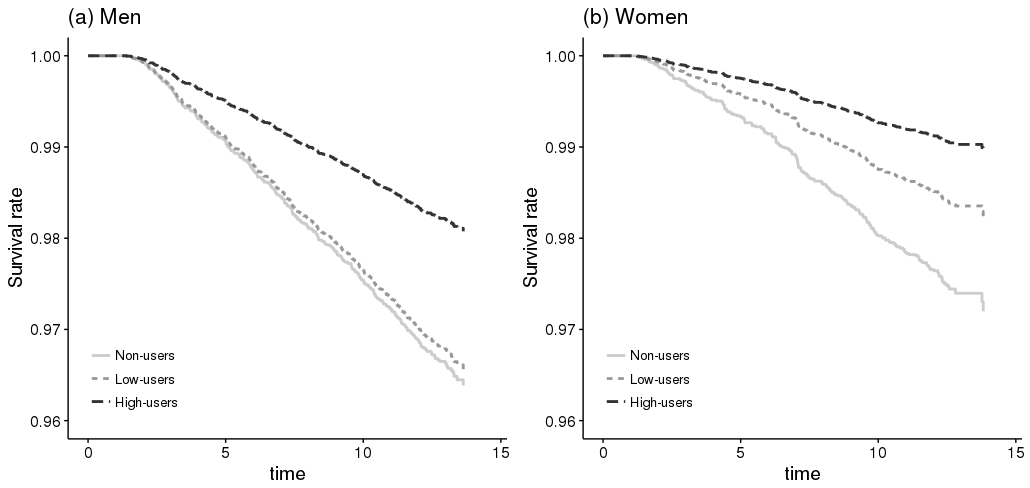

Supplement: Supplementary file 1 [file ijerph-17-01054-s001.zip › ijerph-680701-supplementary.tiff]
